# Roles of Spermatogonial Stem Cells in Spermatogenesis and Fertility Restoration

**DOI:** 10.3389/fendo.2022.895528

**Published:** 2022-05-12

**Authors:** Lei Diao, Paul J. Turek, Constance M. John, Fang Fang, Renee A. Reijo Pera

**Affiliations:** ^1^The First Affiliated Hospital of USTC, Division of Life Sciences and Medicine, University of Science and Technology of China, Hefei, China; ^2^Turek Clinic, San Francisco, CA, United States; ^3^MandelMed, Inc., San Francisco, CA, United States; ^4^McLaughlin Research Institute, Touro College of Osteopathic Medicine – Montana (TouroCOM-MT), Great Falls, MT, United States; ^5^Research Division, Touro College of Osteopathic Medicine – Montana (TouroCOM-MT), Great Falls, MT, United States

**Keywords:** spermatogonia, spermatogenesis, *in vivo*, *in vitro*, stem cell, 3D culture, male infertility

## Abstract

Spermatogonial stem cells (SSCs) are a group of adult stem cells in the testis that serve as the foundation of continuous spermatogenesis and male fertility. SSCs are capable of self-renewal to maintain the stability of the stem cell pool and differentiation to produce mature spermatozoa. Dysfunction of SSCs leads to male infertility. Therefore, dissection of the regulatory network of SSCs is of great significance in understanding the fundamental molecular mechanisms of spermatogonial stem cell function in spermatogenesis and the pathogenesis of male infertility. Furthermore, a better understanding of SSC biology will allow us to culture and differentiate SSCs *in vitro*, which may provide novel stem cell-based therapy for assisted reproduction. This review summarizes the latest research progress on the regulation of SSCs, and the potential application of SSCs for fertility restoration through *in vivo* and *in vitro* spermatogenesis. We anticipate that the knowledge gained will advance the application of SSCs to improve male fertility. Furthermore, *in vitro* spermatogenesis from SSCs sets the stage for the production of SSCs from induced pluripotent stem cells (iPSCs) and subsequent spermatogenesis.

## Introduction

Early in human development, a small group of cells is set aside or allocated to become the germ cells that give rise to the sperm and oocytes that will transmit genetic and epigenetic information to subsequent generations (1). In males, the process of spermatogenesis maintains the production of spermatozoa, the final cell carrier of inheritable material, throughout the lifetime of male mammals (2). Continuous spermatogenesis depends on the appropriate self-renewal and differentiation of spermatogonial stem cells (SSCs) throughout the life of the male (3). The SSCs are the resident stem cell population that resides at the basal membrane of seminiferous tubules of the testis (4, 5). The SSCs can undergo mitotic divisions for self renewal to maintain a steady stem cell pool or they can differentiate through sequential and extensive processes into spermatozoa (6). The balance of self-renewal and differentiation of SSCs is critical, not only for maintaining normal spermatogenesis but also for sustaining lifelong fertility (7). A tilt to self-renewal is a risk factor for germ cell tumors, while a tilt towards differentiation results in exhaustion of germ cell pools, leading to male infertility (8). Numerous studies have demonstrated that the balance between self-renewal and differentiation is precisely controlled by a combination of intrinsic genetic and epigenetic factors within SSCs as well as the extrinsic signals that eminate from the somatic niche (9, 10).

Significantly, SSCs have extraordinary therapeutic potential in assisted reproduction for male infertility (11, 12). Transplantation of SSCs can restore spermatogenesis in patients who suffer from impaired spermatogenesis (13). One application example is fertility preservation of prepubertal boys with cancer and undergoing chemotherapy (14). SSCs can be isolated from testicular biopsy and cryopreserved before chemotherapy, followed by stem cell transplantation into the seminiferous tubules to restore fertility (15, 16). In addition, germline gene therapy using SSCs has been proposed, albeit with obvious concerns regarding legitimate ethical issues, as a promising and feasible approach to treat endocrine disease and metabolic disorders with germline gene mutations (17). Currently, the major hurdle to the use of SSCs in assisted reproductive technology is the difficulty of identificating and isolating endogenous SSCs and directing their differentiation to haploid cells *in vitro*.

This review provides a brief overview summary of some of the existing knowledge and research progress regarding use of SSCs for inducing spermatogenesis *in vivo* and *in vitro* for fertililty restoration. We hope that this summary review may spur further inquiries into details and ongoing studies of practical applications of SSCs in human reproduction and regenerative medicine.

## Regulation of SSCS

Human germ cell development begins with the specification of a small group of cells to form the primordial germ cells (PGCs) (18), which are thought to arise from the dorsal amnion at the onset of gastrulation (19). Following their specification, PGCs actively proliferate and migrate to the developing gonad (20–22) where they will occupy the genital ridge and undergo sex-determination by entering either male or female sex-specific developmental pathways (23). External signals from the somatic environment determine the sex of PGCs (24). For male germ cell development, once PGCs occupy the seminiferous tubules of the male gonad, they are termed gonocytes (25), which later interact with the niche cells to become spermatogonia (26). Note that nomenclature is not universal or all inclusive as subtypes exist (example: type A, type b, light and dark spermatogonia), different stages of development are sometimes indicated (examples: early or late spermagonia or undifferentiated and differentiating), or reference to marker content (example: c-kit+ spermatogonia).

### The Niche

**Figure 1:**
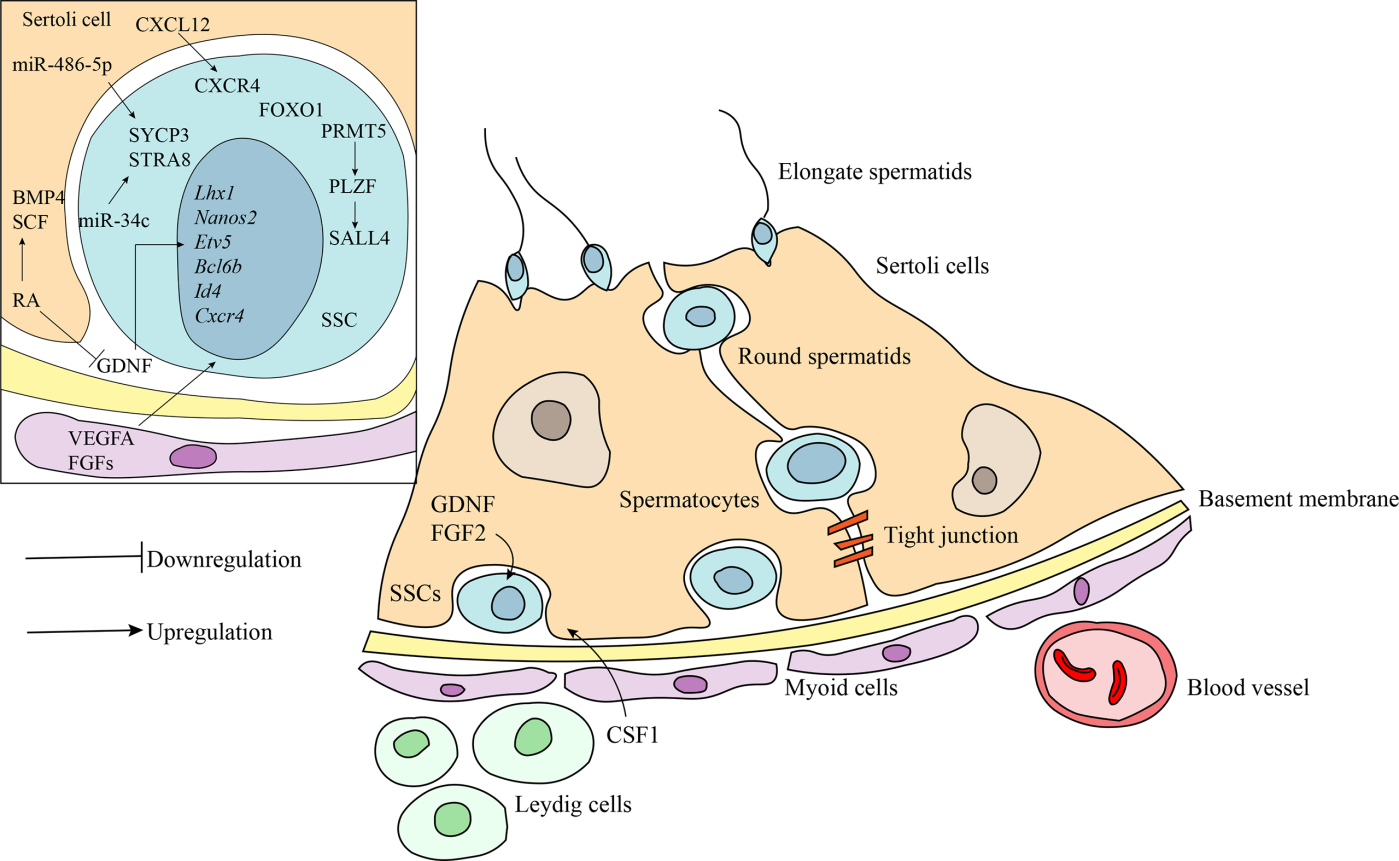
Schematic diagram of the niche of SSCs and the regulatory factors involved in maintaining the stemness and self-renewal of SSCs. Undifferentiated SSCs are localized at the basement membrane. Germ cells maintain the close contact with the Sertoli cells inside the seminiferous epithelium. Peritubular myoid cells surround the seminiferous tubules to form testicular cords. The interstitial compartment consists of many somatic cell types including Leydig cells, mesenchymal cells and immune cells. Bioactive factors in the niche play crucial role in self-renewal and differentiation of SSCs. CXCL12/CXCR4, FGFs, and VEGFA act in synergy with GDNF to maintain SSCs. Retinoic acid (RA) induces the differentiation of SSCs by downregulation, at least in part, of GDNF expression and activation of SCF and BMP4. Transcription factors, PLZF and FOXO1, are involved in regulating SSCs maintenance and spermatogenesis by acting on a subset of downstream target gene. MicroRNAs, including miR-1908-3p, miR-112-5p and miR-31-5p, also act as critical regulators in spermatogenesis.

The architecture of the testes is characterized by two structurally distinct compartments (**Figure 1**), the seminiferous tubule and the interstitial tissue (27). Within the seminiferous tubule, Sertoli cells form a tight blood-testis barrier to divide the seminiferous epithelium into basal and luminal compartments (28). Developing spermatogonia reside on the basal membrane and are further defined by three types of cells: undifferentiated spermatogonia (quiescent SSCs), differentiating spermatogonia (SSCs that undergo active mitosis), and differentiated spermatogonia (29, 30). The Sertoli cells are the supporting cells for the germ cell population in the testes and are essential for maintaining normal spermatogenesis by providing the cellular matrix and by secreting specific growth factors (31). The surrounding interstitial space consists of various cell types that include the Leydig cells, mesenchymal cells, and immune cells, in addition to lymph vessels, nerve fibers, and connective tissues (27). Leydig cells produce the hormone testosterone and cytokines that may function both directly and indirectly to regulate self-renewal of SSCs (32).

### External and Intrinsic Factors

The fine-tuned balance between self-renewal and differentiation of SSCs is regulated by the interplay of extrinsic and intrinsic factors. GDNF, a growth factor produced by the somatic niche cells, is critical for the maintenance of SSCs both *in vivo* and *in vitro* (33). It regulates several essential downstream genes, including the germ cell specific and ubiquitously-expressed genes *Nanos2*, *Etv5*, *Lhx1*, *T*, *Bcl6b*, *Id1*, and *Cxcr4*, to promote SSC self-renewal and inhibit differentiation (34–39). CXCL12/CXCR4 (39), FGFs (33, 40), and VEGF-A (41) act in synergy with GDNF to maintain SSC stem cell status. In contrast, retinoic acid (RA), a hormone secreted primarily by Sertoli cells, plays an indispensable role in inducing differentiation of SSCs by downregulation of GDNF expression and activation of differentiation-promoting factors, such as BMP and SCF (42–45). Genetic ablation studies in mice indicate that several transcription factors are involved in regulating SSC maintenance and recruitment to spermatogenesis. The PLZF transcription factor is expressed by SSCs and interacts with GDNF signaling as one of the master regulators to promote the self-renewal of SSCs (46, 47). Loss of PLZF results in progressive germ cell loss, testicular hypoplasia, and infertility (46–48). One of the downstream targets of PLZF is the SALL4 protein, which is required for the self-renewal of SSCs and maintenance of ability to enter spermatogenic differentiation (49). A potential upstream regulator of PLZF is PRMT5. Disruption of the *PRMT5* gene results in a dramatic reduction of *PLZF* gene expression, and subsequent progressive loss of SSCs leading to male infertility (50). Another transcription factor important for maintenance of SSC self-renewal is FOXO1, which regulates a number of genes that are preferably expressed in SSCs (51). Deletion of the *FOXO1* gene results in defects in SSC maintenance and ultimately spermatogenic failure. In addition, recent research has identified numerous microRNAs as critical regulators in spermatogenesis. Some microRNAs regulate the self-renewal of SSCs. For example, miR-202 plays a crucial role in the maintenance of SSC stemness or self-renewal of the stem cell population (52). Other microRNAs, such as miR-1908-3p (53), miRNA-122-5p (54), and miRNA-31-5p (55), enhance the proliferation and inhibit the early apoptosis of human SSCs *via* targeting key downstream pathways. Conversely, several microRNAs facilitate differentiation *via* regulation of the expression of genes associated with SSC differentiation. MiR-34c promotes SSC differentiation by inhibiting the function of the *NANOS2* gene, leading to the up-regulation of meiotic-related proteins, STRA8, in mice (56). Similarly, miR-486-5p secreted by Sertoli cells stimulates differentiation of SSCs in mice by up-regulating the expression of STRA8 and SYCP3 (57). Further, impaired spermatogenesis is observed in mice carrying a deficiency in miR-17-92 or a gene deletion of miR-17-92 (58, 59). miR-202 similarly regulates spermatogenesis *via* orchestration meiotic initiation by preventing precocious differentiation of mouse SSCs (52). Taken together, numerous genes act to balance self-renewal and differentiation of SSCs.

## Fertility Restoration Through *In Vivo* Spermatogenesis

**Figure 2:**
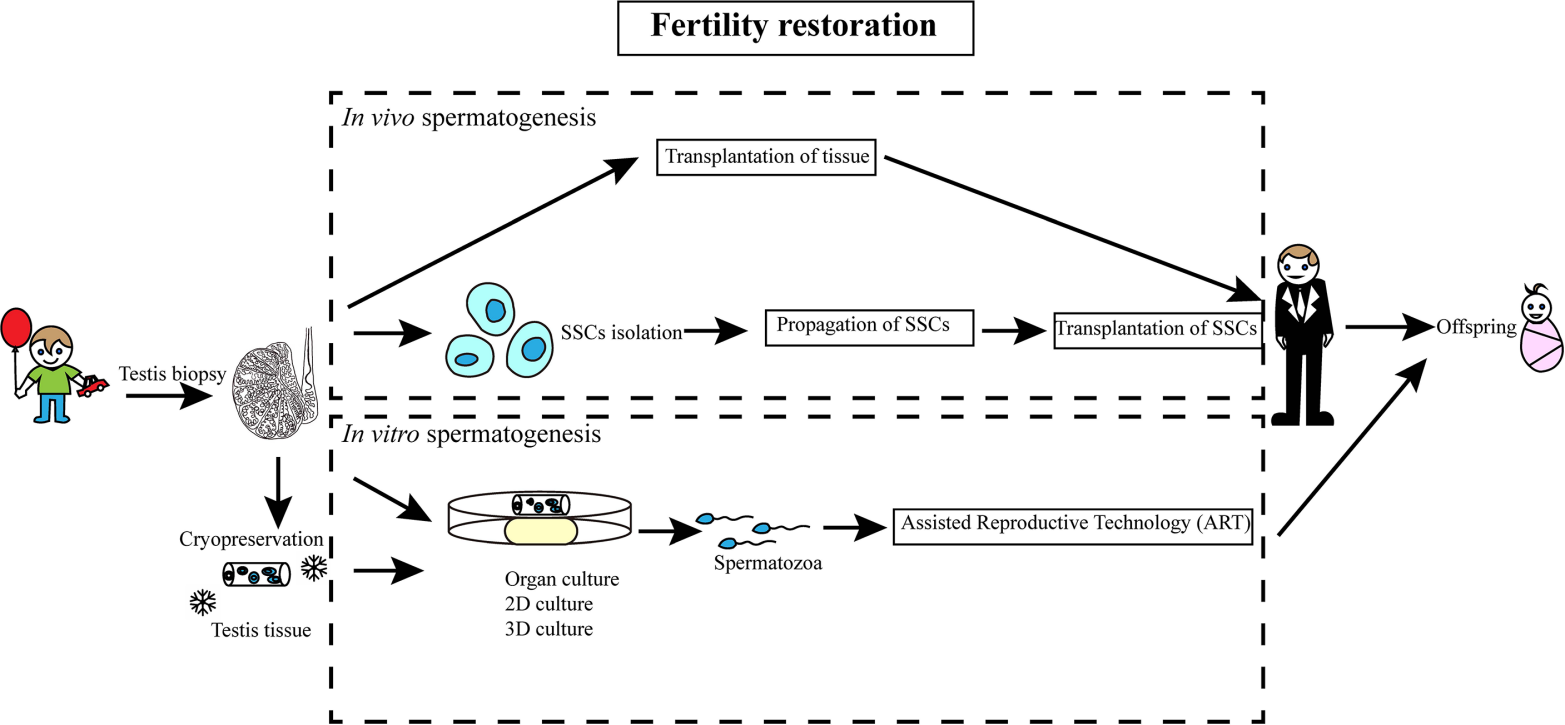
Schematic diagram of SSC-based fertility restoration in humans. A sample of testicular tissue of prepubertal boys, who receive gonadotoxic treatment, is retrieved and cryopreserved. Spermatogenesis may be induced after treatment either *in vivo* or *in vitro*.

SSCs within the testicular tissues have the potential to complete the entire process of spermatogenesis *in vivo* and produce functional spermatozoa for fertility restoration (**Figure 2**). Thus, cryopreservation of testicular tissue prior to gonadotoxic treatment for prepubertal boys is proposed as a helpful strategy for fertility preservation (60). To restore fertility through *in vivo* spermatogenesis, testicular tissues could be either autotransplanted to the same individual or the tissues might be dissociated to obtain SSCs for autotransplantation. Xenotransplantation would carry the obvious complication of mixing of sperm from different individuals.

### Transplantation of Testicular Tissues

Autotransplantation of testicular tissues has achieved success in multiple animal models, which results in live offspring (61–65). However, the approach has the risk of re-introducing malignancy is a concern (66). Studies of xenotransplantation, which transplants immature testicular tissue under the back skin of immune-deficient animals, have been used to examine potential complications including malignancy. In 2002, Nagano and colleagues, for example, transplanted human SSCs into immunodeficient mice for the first time (67). Human SSCs survived in mouse testes for at least six months and proliferated during the first month after transplantation.

### Transplantation of SSCs

To avoid potential complications of malignancy, isolation of SSCs from cryopreserved testicular tissues followed by transplantation has been proposed as the leading alternative stratgey. To separate SSCs from somatic cells, antibodies that recognize human SSC-specific proteins are used for FACS (fluorescent-activated cell sorting) or MACS (magnetic-activated cell sorting) for sorting SSCs from other cell types. Antibodies that have been shown to be useful for sorting SSCs include GFRα (68), GPR125, ID4 (69), ITGA6 (70), SSEA4 (71), PLPPR3 (72), and OCT4 (73). An alternative to cell sorting is to take advantage of different physical properties between SSCs and somatic cells such as velocity sedimentation and differential affinity to extracellular matrices on the culture plate (74–78). Once isolated, SSCs are cultured with growth factors shown to be optimal or essential for SSC maintenance [GDNF, BFGF, EGF, and LIF (79–81)].

A major limitation of SSC transplantation *in vivo*, for fertility restoration in clinical practice, is the scarcity of SSCs within the testicular tissue. This has necessitated exploration of alternatives including the establishment of a robust *in vitro* culture system to maintain and expand human SSCs. Extensive effort has been focused on optimization of culture conditions for long-term maintenance and propagation of human SSCs. Multiple culture substrates, including hydrogel, matrigel, and laminin, have been shown to promote the propagation of human SSCs under feeder-free conditions (82). Currently, several markers are used for the verification of human SSCs. However, many of these markers are also expressed in testicular somatic cells. For example, UCHL1, which was used to identify SSCs from humans, is also expressed in Leydig cells and nerve fibers (83). The most stringent assay to assess the function of SSCs is to generate offspring after homologous transplantation. However, despite success in animal models, including non-human primates, no studies are reporting the generation of human functional spermatozoa following autotransplantation or xenotransplantation of testicular tissue or isolated human SSCs for fertility restoration.

## Fertility Restoration Through *In Vitro* Spermatogenesis

The establishment of a system to recapitulate spermatogenesis and generate spermatozoa *in vitro* can not only be directly applied in assisted reproduction, such as *in vitro* fertilization (IVF) or intracytoplasmic sperm injection (ICSI), but also provide a convenient system to study the molecular mechanisms and genetic causes for male infertility. Building a functional somatic microenvironment is critical for *in vitro* spermatogenesis. Several strategies, including exploitation of intrinsic somatic microenvironment by organotypic culture, two-dimensional culture, and three-dimensional culture of testis cell suspensions.

### Organotypic Culture

Since 1959, a gas-liquid interface was used to culture testicular fragments of the adult rats (84). In this culture system, the differentiation of SSCs was limited up to pachytene spermatocytes (85). In 2003, round spermatids were observed after two weeks of culture in a gas-liquid interface culture system (86). Several other organotypic culture systems have been developed to recapitulate the entire process of spermatogenesis *in vitro*. One of the breakthroughs in the research was reported in 2011 with the demonstration of live offspring that were generated from *in vitro*-produced haploid germ cells (87). In this study, testicular tissue fragments from neonatal mice were cultured on an agarose gel-based organ culture system. Subsequently, microfluidic technology was adopted for organ culture, with the goal of providing a better culture environment for SSCs by facilitating the exchange of gases, nutrients, and waste products (88). Recently, successful recapitulation of human testicular organogenesis from fetal gonads was achieved, and *in vitro*-derived haploid spermatids were shown to undergo meiotic recombination (89).

### Two-Dimensional Culture

2D culture systems with testis cell suspensions have been widely used for SSC proliferation and differentiation with two primary types of 2D culture systems most common: (1) SSCs cultured on mitotically-inactivated feeder cells, (2) SSCs co-cultured with somatic cells (90). Using the support of 2D culture sytems, numerous studies have reported that haploid male germ cells could be induced (91–95), and offspring can be produced from these *in vitro* derived haploid male germ cells in rodent (96). However, the 2D culture system has not been optimized for human germ cells. This may be due to the lack of spatial structure of seminiferous tubules and proper interactions between germ cells and somatic cells.

### Three-Dimensional Culture

To better mimic the testicular niche, various 3D culture systems have been developed. In 2006, testicular cells isolated from rats were cultured on collagen gels to mimic the composition of the basal membrane of seminiferous tubules (97). Later, the soft-agar culture system (SACS) was developed (98), and mice haploid germ cells from undifferentiated germ cells were generated in this system in 2012 (99). The SACS system also supports the differentiiation of SSCs of non-human primates. The most commonly used alternate material in 3D culture system is methylcellulose. The methylcellulose culture system (MCS) also supports the differentiation of immature germ cells.

In order to artificially reproduce the *in vivo* form and function of the seminiferous epithelium, a 3D engineered blood-testis barrier (eBTB) system was designed in 2010 (100). Testicular peritubular myoid cells were first cultured on the underside of culture inserts, and then germ cells and Sertoli cells were added on top of the inserts. The testicular cells from neonatal mice form the aggregate by culturing on a V-shaped plate. The aggregate plated on the top of agarose gel blocks, and the haploid male germ cells were obtained after 30-51 days of incubation (101).

The 3D decellularized testicular scaffold with hyaluronic acid and chitosan provides the condition for the differentiation and proliferation of mice SSCs (102). The proliferation and self-renewal of mice SSCs was stimulated by culturing on the 3D scaffold consisting of alginate hydrogel with Sertoli cells (103). The mice germ cells were cultured in 3D printed one-layer scaffolds at the air-medium interface simulating the tubule-like structure. This culture system provided the condition for long-term survival and differentiation (104).

Soft agar and agarose gel are the most common material used to establish the 3D culture system for human SSCs. A soft agar culture system has been shown to support the proliferation and differentiation of human SSCs (105). Another material that has been used in 3D culture systems for human SSCs is a polycaprolactone (PCL) nanofiber matrix (106). This material may mimic the physical form of collagen fibers in the natural extracellular matrix (107).

## Conclusion and Perspectives

With the development of technologies, including -omics at the single-cell level, lineage-tracing, spermatogonial transplantation, and *in vitro* culturing and differentiation, we start decoding the secrets of SSCs. However, the application of SSCs to treat male infertility necessitates extensive studies to ensure safety and efficacy. An efficient culture condition for human SSCs to ensure their propagation, as well as proper animal models for xenotransplantation, will assist in assessing safety and efficacy as indicated by recent studies (108). Furthermore, establishing a robust system for *in vitro* spermatogenesis is also helpful for pharmaceutical or toxicological studies for new drugs. Finally, *in vitro* spermatogenesis from SSCs sets the stage for the production of SSCs from induced pluripotent stem cells (iPSCs) and subsequent spermatogenesis. For example, studies are underway to integrate data and practices from divergent fields to promote spermatogenesis from iPSCs *via* co-culture with Sertoli cells in a 2D-, 3D- or a modified environment, similar to those used in other physiological systems, that might more faithfully mimic spermatogenic dynamics including circulation (109, 110).

## Author Contributions

All authors contributed to the article and approved the submitted version.

## Funding

This work was supported by an NIH grant to RRP #HD096026 and National Natural Science Foundation of China #32070830 to FF.

## Conflict of Interest

Authors PJT and CMJ are founders of the company MandelMed. No funding from MandelMed is associated with this study.

The remaining authors declare that the research was conducted in the absence of any commercial or financial relationships that could be construed as a potential conflict of interest.

## Publisher’s Note

All claims expressed in this article are solely those of the authors and do not necessarily represent those of their affiliated organizations, or those of the publisher, the editors and the reviewers. Any product that may be evaluated in this article, or claim that may be made by its manufacturer, is not guaranteed or endorsed by the publisher.
